# Intracanal placement of calcium hydroxide: a comparison of specially designed paste carrier technique with other techniques

**DOI:** 10.1186/1472-6831-13-52

**Published:** 2013-10-07

**Authors:** Joseph Meng Ern Tan, Abhishek Parolia, Allan Kah Heng Pau

**Affiliations:** 1 School of Dentistry, International Medical University, Bukit Jalil, Kuala Lumpur, 57000, Malaysia; 2 Faculty of Dentistry, International Medical University, Bukit Jalil, Kuala Lumpur, 57000, Malaysia

**Keywords:** Root canal therapy, Intracanal medicament, Calcium Hydroxide, Placement techniques

## Abstract

**Background:**

This study compared the effectiveness of a Specially Designed Paste Carrier technique with the Syringe-Spreader technique and the Syringe-Lentulo spiral technique in the intracanal placement of calcium hydroxide.

**Methods:**

Three groups, each containing 15 single-rooted human anterior teeth were prepared using standardized Mtwo rotary instruments to a master apical file size 40 with 0.04 taper. Each group was filled with calcium hydroxide paste using: Syringe and #25 finger spreader (Group 1); Syringe and #4 rotary Lentulo spiral (Group 2), Specially Designed Paste Carrier (Group 3). Using pre-filling and post-filling radiographs in buccolingual and mesiodistal planes, the radiodensities at 1 mm, 3 mm, 5 mm, and 7 mm from the apical foramen were analyzed by ANOVA and Bonferroni post hoc tests.

**Results:**

Overall, The Specially Designed Paste Carrier technique showed a statistically significantly higher mean radiodensity than the two other compared techniques. No significant difference was detected between the Syringe-Lentulo spiral and the Syringe-Spreader techniques.

**Conclusion:**

The Specially Designed Paste Carrier technique was more effective than the Syringe-Spreader technique and the Syringe-Lentulo spiral technique in the intracanal placement of calcium hydroxide.

## Background

As a consequence of pathological changes in the dental pulp, the root canal system can harbor numerous irritants. Egress of these irritants from the infected root canals into the surrounding tissues can initiate the formation and perpetuation of peri-radicular lesions. Studies have indicated that the main irritants to cause pulpal and periradicular lesions are micro-organisms [1,2]. Therefore, the primary objective of endodontic therapy is to reduce or eliminate microorganisms and their by-products from the root canal system. This can be achieved to a great extent by thorough chemo-mechanical debridement. Although a number of instrumentation and irrigation techniques exist, complete debridement is impeded due to the complex anatomy of the root canal system and the consequent limitations of access by instruments as well as irrigants [3]. Thus the use of antimicrobial intra-canal medication has been advocated to disinfect the root canal system [2,4]. Calcium hydroxide is acknowledged as one of the most effective intra-canal medicaments used in endodontics due to its bactericidal properties [5]. The specific mechanism of action of calcium hydroxide is still a matter for debate [2,6]. Some researchers suggest that this antimicrobial activity is due to the release and diffusion of hydroxyl (OH-) ions leading to a highly alkaline environment (pH 12.5-12.8) which is non-conducive to the survival of micro-organisms [2,6,7].

It has been shown that calcium hydroxide kills microorganisms by direct contact, and hence it should ideally occupy the canal space with maximal density and depth to the working length in order to permit its biological effects to be exerted in closest proximity to the appropriate tissues [8].

The techniques for intracanal calcium hydroxide placement have been investigated within in-vitro studies, providing varying results in terms of effectiveness in filling root canals(as summarized in Table 1) [9-15]. This study aims to evaluate the extent and fill-density of calcium hydroxide placement in the root canal with a Specially Designed Paste Carrier and to compare it with other commonly employed techniques such as the Syringe-Lentulo spiral and the Syringe-Spreader techniques in the intracanal placement of calcium hydroxide.

**Table 1:** Summary table of previous research studies

**Study**	**Canal type/MAF #**	**Comparison of techniques**	**Main findings/conclusions**
Estrela et al [9].	Dog Premolars, #50	**Ca(OH)2 placement by:** Endodontic file/ McSpadden Compactor/ Lentulo	Endodontic file was superior.
Deveux et al [10].	Single-rooted human premolars, #25	**Ca(OH)2 placement by:** MecaShaper/ K-type ultrasonic file/ Gutta-Condensor, Pastinject/ Lentulo	Pastinject was superior.
Torres et al [11].	Simulated 44° curved canal, #40	**Ca(OH)2 placement by:** Ultradent tip/ Lentulo/ Ultradent + Lentulo (Combined)	1 mm (from terminus): Lentulo was superior. 3 mm: Lentulo and combined were superior.
Oztan et al [12].	Simulated 42° curved canal, #40	**Ca(OH)2 placement by:** Lentulo/ Pastinject **Ca(OH)2 vehicles:** Glycerin/Water (Calcium Hydroxide was mixed with either glycerin or water and placed with either Lentulo or Pastinject.)	Glycerin was superior as a vehicle of Ca(OH)2 for Pastinject or Lentulo. Pastinject was superior to Lentulo with either vehicles of Ca(OH)2.
Simcock et al [13].	Single-canal Human 2^nd^ Mandibular Premolars*	**Ca(OH)2 placement by:** Lentulo/ injection system/ Flex-O file/ Reverse rotary NiTi Canals were minimally prepared. (MAF 25) or completely prepared (MAF 40).	Completely prepared canals had fewer voids for all placement techniques. Injection system was superior in completely prepared canals
Peters et al [14].	Simulated 50° curved canal*	**Ca(OH)2 placement by:** Lentulo/ injection system **Canals were prepared to MAF: #**20/#30/#40	Lentulo was superior. MAF #40 canals had fewest voids.
Deonizio et al [15].	Single-canal Human Mandibular premolars, #50	**Ca(OH)2 placement by Lentulo with speed:** 5000 rpm, 10000 rpm, 15000 rpm	Varying speeds are needed for optimal Ca(OH)2 filling. 15000 rpm was superior in apical third. 5000 rpm was superior in filling middle and cervical thirds.

*indicates studies with varying MAF (Master Apical File) sizes for the sake of comparison.

## Methods

Forty five fully developed, single rooted human anterior teeth (incisors and canines) were selected from a pool of teeth collected by request from the Cahaya Suria government dental clinic in Kuala Lumpur, Malaysia. According to the Malaysian Ministry of Health Guidelines For Ethical Review of Clinical Research or Research Involving Human Subjects (2006), studies involving biological specimens with no interaction with the human subjects involved; and with no collection of any identifiable private information are automatically exempted from obtaining informed consent from study subjects. This study was approved by the Research and Ethics committee of the International Medical University, Bukit Jalil (Project approval number: BDS I01/2009(02)2012).

The selected teeth were cleaned by ultrasonic scaler to remove the attached calculus and debris. The teeth were stored in 0.5% Chloramine T Trihydrate solution for between two to four weeks until use. Each tooth was between 21-26 mm in total length from incisal edge or canine cusp tip to anatomical apex. Following occlusal access of the pulp chamber with carbide round burs of ISO size 010 and Endo-Z bur FG ISO 016 (Dentsply Maillefer, Ballaigues, Switzerland), the working lengths were determined by visualizing the tip of a #10K-file through the apical foramen of each tooth and subtracting 1 mm from the length of the file. Canal instrumentation was performed using Mtwo rotary Nickel titanium endodontic instruments, VDW Silver motor and handpiece (VDW, Munich, Germany) according to the manufacturer’s specifications until a standardized master apical file (MAF) size 40, 0.04 taper. Canals were irrigated with 1.0% sodium hypochlorite and patency was ensured by placement of #10 K-file (Mani, Japan) in between placement of each file. A final flush of saline was made. After canal shaping and cleaning, the canals and pulp chambers were dried with cotton pellets and #40 paper points (Dura, Japan). The teeth were then mounted with Aquasil Soft putty (Dentsply, DeTrey GmbH, Germany) into an arch form simulating a dental arch. Each group of teeth was mounted into one arch, resulting in 3 arches. These teeth (n=45) were randomly divided into three groups according to the technique employed to dress the root canal with a ready-mixed paste of calcium hydroxide containing barium sulphate as a radioopaquer (Calcicur, Voco GmbH, Germany) as follows:

Group I- Root canals were filled using the Calcicur syringe and needle tip (Voco GmbH, Germany) provided by the manufacturer and by counter-clockwise rotation of a # 25 finger spreader. These steps were repeated until the paste extruded from the canal orifice.

Group II- Root canals were filled using the Calcicur syringe and needle tip (Voco Gmbh, Germany) provided by the manufacturer followed by placement of a Lentulo spiral in a slow speed handpiece. A #4 Lentulo spiral RA 25 mm (Dentsply Maillefer, Ballaigues, Switzerland) was selected by placing it passively up to 1 mm short of the working length before dressing the root canal with the paste. Thereafter, the whole length of the Lentulo spiral was coated with the calcium hydroxide paste and inserted up to 1 mm from working length. The procedure was repeated until the paste extruded from the canal orifice indicating adequate fill.

**Figure 1:**
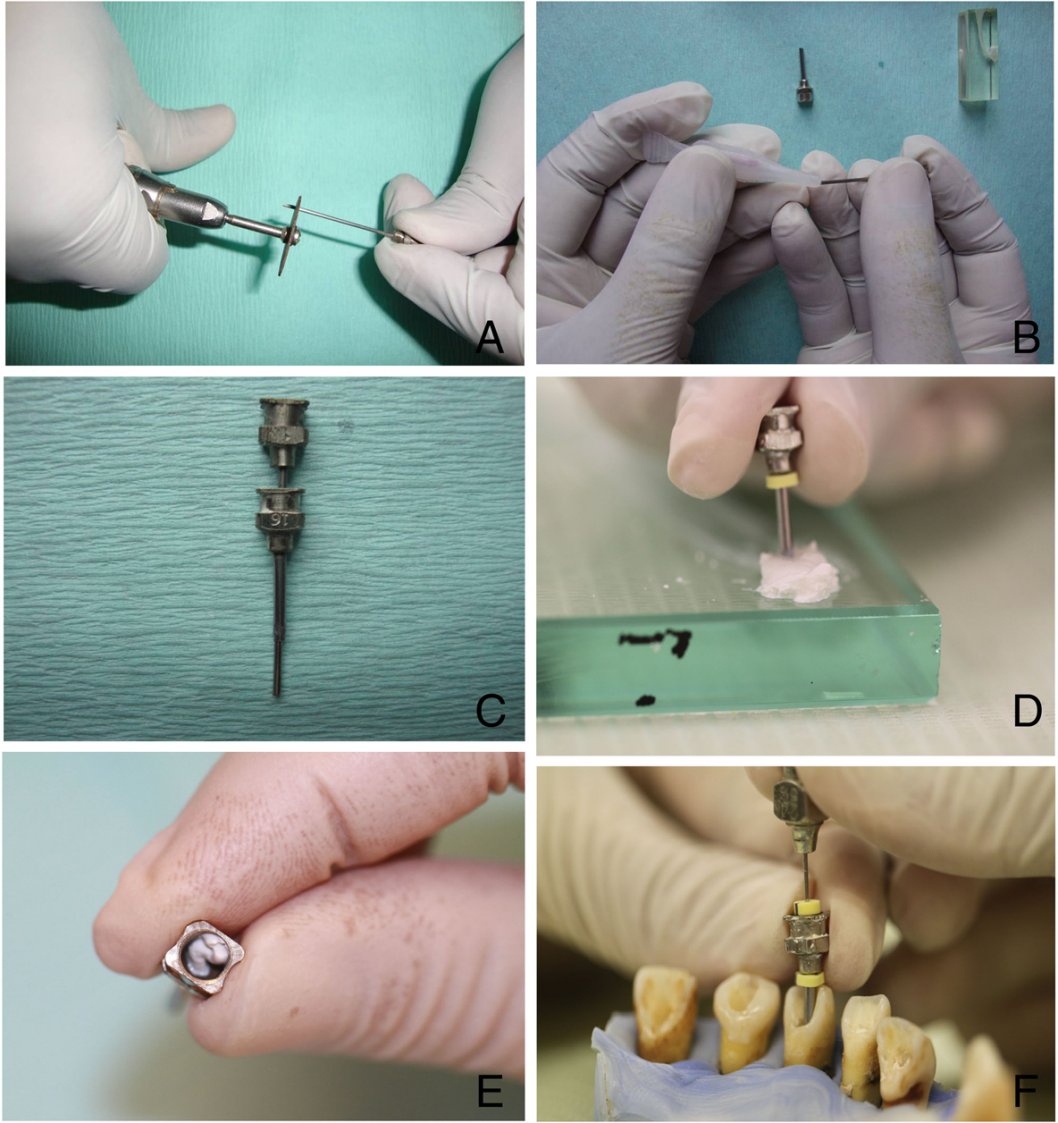
**Methodology of the specially designed paste carrier technique. (A)** Sectioning of needle with a diamond disc. **(B)** Lumen of thinner needles (18, 21 and 24 gauge needles) sealed with cyanoacrylate. **(C)** Checking for smooth fit and movement of thinner needles through the patent 16 gauge needle. **(D)** Tapping the 16 gauge needle on the mixed calcium hydroxide paste. **(E)** The excess calcium hydroxide paste extruding from the opening of the needle hub. **(F)** Compaction of the paste into the canal with the length adjusted stoppers on the needles.

Group III- Root canals were filled using the Specially Designed Paste Carrier (SDPC) technique as follows:

Four reusable sterile stainless steel luer-lock needles (Doctor, India) of 16 (1.29 mm diameter), 18 (1.02 mm diameter), 21(0.72 mm diameter) and 24 gauge (0.51 mm diameter) were selected. The largest diameter (16 gauge) needle was sectioned with a diamond disc (Figure 1A) leaving 10 mm measured from tip to the hub of the needle. The smaller diameter needles were sectioned with a diamond disc to remove just the bevel of the needle. The lumen of the thinner needles (18, 21 and 24 gauge) which were used as pluggers were sealed carefully with cyanoacrylate to block only the lumen (Figure 1B), any excess cyanoacrylate on the outer surface of the needles were cleaned off. The thinner needles were inserted into the thickest (16 gauge) needle one at a time to check for fit and smooth movement (Figure 1C). Rubber stoppers were adjusted on the thickest (16 gauge) and thinnest (24 gauge) needle up to 2 mm short of the working lengths measured with both needles positioned in place at the canal orifices.

The calcium hydroxide paste (Calcicur, Voco GmbH, Germany) was placed on a glass slab and mixed with a chemically pure calcium hydroxide powder (Produits Dentaires SA, Vevey, Switzerland) at a 2:1 paste to powder ratio to produce a thicker consistency of paste. The thickest needle (16 gauge) was tapped on the calcium hydroxide paste to fill the lumen of the needle from the tip upwards until the paste extruded from the opening of the needle hub (Figure 1D and E). The needle filled with calcium hydroxide was then placed at the orifice of the root canal and held between the thumb and middle finger. The thinnest diameter (24 gauge) needle was used as a plugger to deliver the mass of calcium hydroxide into the root canal in an up-down motion up to the length of the measured stopper (Figure 1F). This procedure was repeated with the other needles (21 gauge and 18 gauge) in sequential order and by decreasing the length of the needle gradually as the canal filled up coronally until the calcium hydroxide paste finally extruded from the canal orifice.

Immediately after placement of the calcium hydroxide paste into the root canals of each group of teeth, the excess paste was cleaned from the orifice and a restoration was placed to seal the orifice of the canal using IRM (Dentsply Caulk, USA) according to the manufacturer’s instructions.

**Figure 2:**
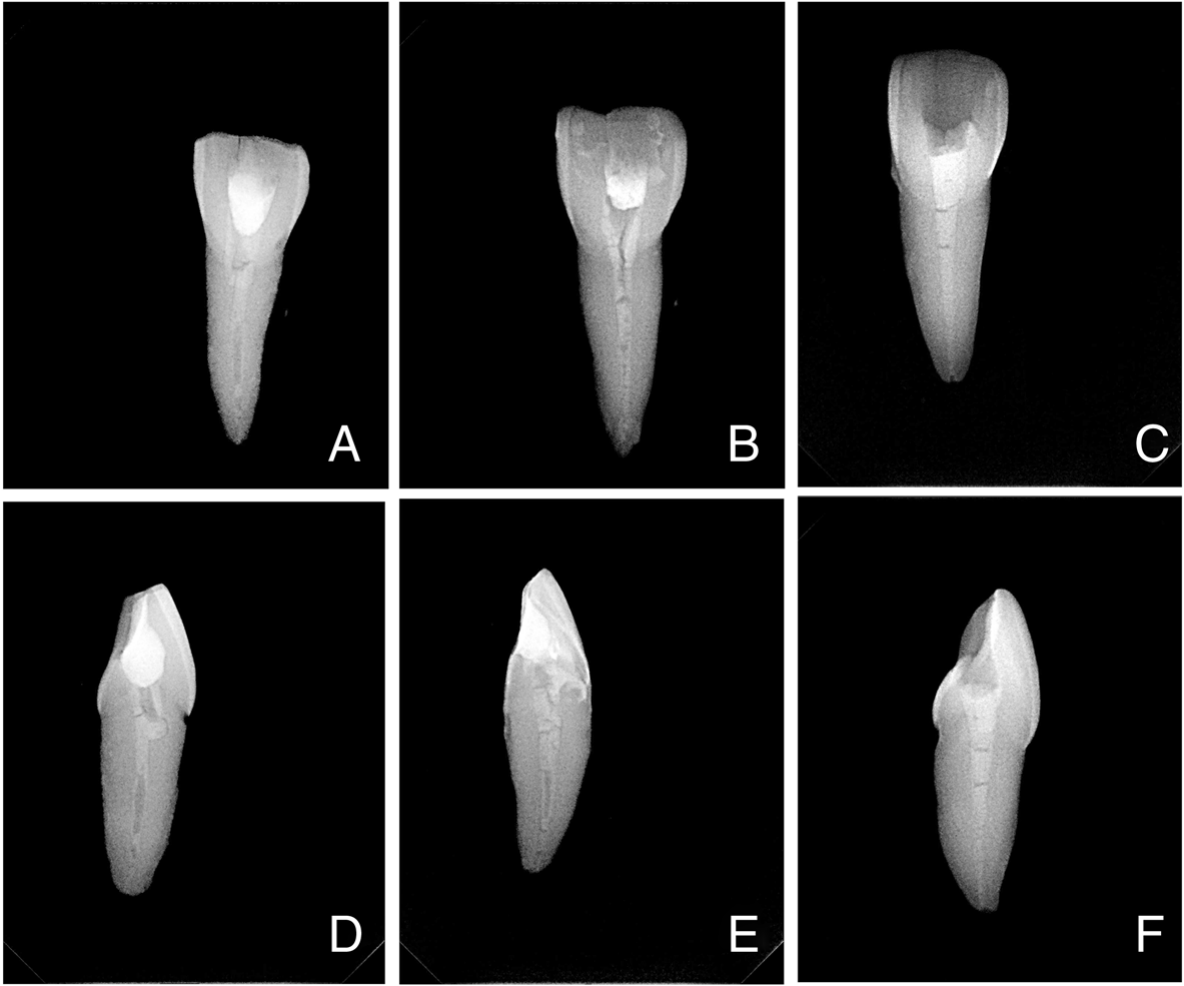
**Sample radiographic images (buccolingual and mesiodistal views) of teeth from each of the 3 groups. A** and **D**: Syringe-Spreader Technique; **B** and **E**: Syringe-Lentulo Spiral Technique; **C** and **F**: Specially Designed Paste Carrier Technique.

The teeth were radiographed prior to mounting in the silicone putty in both buccolingual and mesiodistal planes using the Gendex Oralix AC and Visualix eHD digital intraoral sensor (Gendex Dental Systems, Des Plaines, IL, USA). The head of the X-ray tube was positioned at a distance of 10 cm and at a 90° angle from the plane of the sensor. The sensor was held by a cardboard positioning device and each tooth was taped to the sensor to ensure that all radiographs were taken from the same position with an exposure time of 0.10 seconds. The teeth were removed from the silicone putty after filling with calcium hydroxide paste and IRM placement, and radiographed in the same manner as before. A total of four radiographs (before and after filling) were thus taken for each tooth. A sample of post-filling radiographs of teeth from each of the three groups is displayed in Figure 2. Each image was captured in a digital format with a resolution of 25.6 line pairs/mm into the VixWin Platinum software (Gendex Dental Systems, Hatfield, USA). Calibration was performed and measurement annotations were made on each image using the VixWin ruler function at 1.0 mm, 3.0 mm, 5.0 mm, and 7.0 mm (±0.1 mm) from the apical foramen of each tooth. Density values were measured using the VixWin gray section whereby a line was drawn at two points at the center of the canal and measurements were made at specific points on the line corresponding to the aforementioned measured lengths. The VixWin gray section consists of a scale of 0–256 shades of gray; the greater the radio-opacity, the higher the gray section reading. The difference in density measurements before and after placement of the calcium hydroxide paste was obtained.

The mean of the buccolingual and mesiodistal image density measurements were calculated at each level of the canal for each tooth. The data was entered into SPSS version 18.0 for analysis. The mean radiodensity values of each group of teeth with the 95% confidence intervals at each level are presented in Table 2. The one way ANOVA and Bonferroni post hoc tests were performed to test for statistically significant differences between the three different techniques at each level.

**Table 2:** ANOVA and Bonferroni post hoc test* comparing mean radiodensity values according to technique and depth

**Mean radio-density**	**Technique**			p-values*		
At depth of	1 (Syringe Spreader)	2 (Syringe Lentulo Spiral)	3 (Specially Designed Paste Carrier)	1 cf 2	1 cf 3	2 cf 3
1 mm	6.9 (3.2-10.5)	13.9 (6.6-21.1)	28.8 (22.6-35.0)	0.236	0.001	0.001
3 mm	14.1 (7.1-21.2)	23.9 (17.6-30.2)	35.2 (28.9-41.6)	0.086	0.001	0.037
5 mm	13.5 (7.1-19.8)	21.9 (12.7-31.1)	36.8 (31.3-42.2)	0.250	0.001	0.009
7 mm	22.4 (16.7-30.2)	23.5 (15.5-31.4)	34.5 (28.5-40.6)	1.000	0.047	0.079
Overall density	14.2 (9.9-18.6)	20.8 (16.0-25.6)	33.83 (30.2-37.5)	0.077	0.001	0.001

Mean Radiodensity in Vixwin Gray Section Values and 95% Confidence intervals in brackets.

*refers to the p-value obtained from the post hoc test as indicated in the p-value heading with the same superscript.

## Results

The radiodensity scores for each group are displayed in Table 2. The overall radiodensity score was statistically significantly higher when using the SDPC compared to using SS (p=0.001) or SLS (p=0.001). No statistically significant difference was noted between SS and SLS (p=0.077). At each level, statistically significantly higher radiodensity scores were noted when using the SDPC compared to the SS. Similarly, statistically significantly higher radiodensity scores were noted when using the SDPC compared to the SLS except at the level of 7 mm from the apex. No statistically significant differences were noted between SS and SLS at all levels.

## Discussion

Calcium hydroxide may be delivered into the root canal through a variety of methods, including the use of syringe, rotary instruments such as the Lentulo spiral and hand instruments such as spreaders and pluggers. In this study, the Specially Designed Paste Carrier was compared with two established techniques, the Syringe-Lentulo spiral and the Syringe-Spreader techniques, both of which are used commonly. The degree of canal preparation was found to affect optimal placement of calcium hydroxide in previous studies, thus in the present study, each tooth was prepared up to a standardized MAF size 40 as recommended by Peters et al and Simcock et al [13,14].

The Specially Designed Paste Carrier has shown better results due to the fact that it utilizes a large diameter needle that acts as a 'barrel’, to enable calcium hydroxide paste of a dense and thicker consistency to be directed more effectively into the canal. This minimizes the excess material at the orifice of canals that may otherwise obstruct and prevent further paste condensation. The use of needles with increasing diameter ensures optimal calcium hydroxide paste condensation in conjunction with the increasing canal diameters coronally. The use of length adjusted stoppers allows accurate condensation of the paste to working length while preventing extrusion beyond. This novel method is cost effective as the luer-lock needles are reuseable and can be autoclaved.

Estrella et al postulated that calcium hydroxide paste with a consistency exceeding that of toothpaste is ideal for placement with hand instruments but not for rotary instruments such as the Lentulo spiral [9]. The investigators have found that the Specially Designed Paste Carrier performed better with a paste of thicker consistency during their pilot study, where they have empirically adjusted the consistency of the Calcicur paste by proportioning a 2:1 paste/powder ratio. This resulted in a dense and homogeneous intracanal filling. However, the same thick consistency of paste resulted in more voids and poorer results in canal fillings when employing the other two techniques. Hence, the recommended readily mixed consistency of the Calcicur paste was used for the Syringe-Lentulospiral and Syringe-Spreader methods. The Calcicur paste is a ready-to-use water based paste consisting of: 45% calcium hydroxide, cellulose derivatives and barium sulphate as a radio-opaquer. A chemically pure calcium hydroxide powder was used with the Specially Designed Paste Carrier without the addition of any radio-opaquer. The resultant differences in radiodensity of a canal filling are therefore directly dependent on the quantity of calcium hydroxide contained in the canal as well as the presence or absence of voids.

Various methods have been used to evaluate the quality and extent of intracanal calcium hydroxide fillings in previous studies [9-16]. Differences in weight measurements before and after canal filling have been significantly correlated to radiographic filling appearance [13]. However, weight alone does not give an accurate interpretation of filling quality at specific levels of the canal since it is only a reflection of overall filling density. Observer evaluation methods tend to be subjective in nature as they rely on the visual perception of individuals. Digital image analysis on the other hand presents a more objective and measureable option for the purpose of accurate intracanal filling evaluation [10]. The VixWin gray section digital image analysis used in this study was validated and employed by Torres et al for the measurement of calcium hydroxide filling densities in simulated root canals [11]. Due to the 2 dimensional limitations of a single radiograph, radiographs were taken in both buccolingual and mesiodistal planes. By subtracting the radiodensity value prior to filling from the post-filling radiodensity value, the variations in mineralized tissue densities were thus accounted for [10].

The Syringe-Lentulo spiral group within this study showed a greater mean radiodensity than the Syringe followed by #25 finger spreader group at all levels although no significant difference was detected between both groups. This result is comparable to that of Siggurdsson et al, who found that the Lentulo Spiral performed better than the use of the syringe followed by #25 finger plugger in the delivery of calcium hydroxide paste into the root canal. However, no statistical analysis was performed in the study by Siggurdsen et al [16]. The mean radiodensity was least at the apical 1 mm as compared to the more coronal regions for all 3 techniques. The same trend was discovered by Deveux et al. This may be due to the difficulty of filling the narrow apical third [10]. However, it should also be noted that the difference in canal widths between the apical and coronal regions may result in a disproportionately greater volume of calcium hydroxide coronally giving rise to the greater radiodensity values.

## Conclusion

Within the limits of this in-vitro study, it is concluded that the Specially Designed Paste Carrier was more effective than the Syringe-Lentulo spiral and Syringe-Spreader techniques in the delivery of calcium hydroxide into the canals of single-rooted human anterior teeth, with greater radiodensity of filling up to the working length.

### Limitations and recommendations for future research

Further comparisons of the Specially Designed Paste carrier may also be made with other techniques, with different types of teeth and canal configurations, or with other consistencies and vehicles of calcium hydroxide paste. Clinical trials should be performed to ascertain the effectiveness of this technique in clinical practice settings. In the case of curved canals, flexible nickel titanium needles or instruments may be used as opposed to the more rigid stainless steel needles used in this study. A limitation of this method may be its use in severely curved canals, whereby the access of needles may be limited. The concepts derived from this study may be applied in the manufacture of instruments for greater precision and effectiveness in the delivery of intracanal medicaments.

## Abbreviations

MAF: Master apical file; SS: Syringe-spreader; SL: Syringe-lentulo spiral; SDPC: Specially Designed Paste Carrier; JMET: Joseph Meng Ern Tan; AP: Abhishek Parolia; AKHP: Allan Kah Heng Pau.

## Competing interests

The authors declare that they have no competing interests.

## Authors’ contributions

JMET performed the endodontic preparation of teeth, placement of calcium hydroxide paste by the various techniques, radiographic evaluation and participated in refining the design of the study. JMET drafted the manuscript and participated in the statistical analysis of results. AP conceived the main design of the study, coordinated and supervised the implementation and acquisition of funding for the study. AP also contributed significantly to the preparation of the manuscript and in its review. AKHP refined the design of the study, tabulated the data, performed the statistical analysis and systematic interpretation of the results. AKHP also reviewed the manuscript. All authors have read and approved the final manuscript.

## Authors’ information

JMET is a fifth year Bachelor of Dental Surgery (BDS) student at the School of Dentistry, International Medical University, Bukit Jalil, Malaysia. AP graduated with honours (BDS Hons) from Manipal University, India, and received his postgraduate training from the same institute where he acquired academic and clinical training in Restorative Dentistry and Endodontics (MDS Endodontics and Restorative Dentistry). He has published and reviewed many papers in international scientific dental journals. He has special interests in the field of endodontic microbiology and bioactive materials. He is currently a senior lecturer and endodontist at the School of Dentistry, International Medical University. AKHP graduated with BDS from King’s College London and received his MSc and PhD from Queen Mary University of London. He was elected to The Royal College of Surgeons of Edinburgh as a Fellow in Dental Surgery (FDS) upon successful completion of his specialist training in Dental Public Health, and was entered into the UK’s General Dental Council List of Dental Public Health Specialists. He is currently Professor and Director of the Oral Health Center, International Medical University.

## Pre-publication history

The pre-publication history for this paper can be accessed here:

http://www.biomedcentral.com/1472-6831/13/52/prepub
